# Degradation Behavior of Coated Metallic Stents: Influence of In Vitro Fluid-Dynamic Biostability Testing Conditions

**DOI:** 10.3390/ma18010046

**Published:** 2024-12-26

**Authors:** Muhammad Saqib, Natalia Beshchasna, Gianaurelio Cuniberti, Joerg Opitz

**Affiliations:** 1 Fraunhofer Institute for Ceramic Technologies and Systems IKTS, Maria-Reiche-Strasse 2, 01109 Dresden, Germany; joerg.opitz@ikts.fraunhofer.de; 2 Institute of Materials Science and Max Bergmann Center of Biomaterials, Technische Universität Dresden, 01062 Dresden, Germany; g.cuniberti@tu-dresden.de

**Keywords:** stent, titanium oxynitride, deposition, degradation product, coated stent, bare metal stent, fluid dynamic test

## Abstract

Coated metallic stents are the next generation of metallic stents with improved surface properties. To evaluate the degradation behavior of stents in vitro, different in vitro degradation models can be applied: (i) static immersion test: degradation under static fluid condition, (ii) fluid dynamic test: degradation under flowing fluid, and (iii) electrochemical corrosion test: degradation under the influence of electric potential. During these experimental procedures, stents interact with the simulated blood plasma, and degradation products are formed in the form of depositions on the stent surface, likewise in vivo experiments. These deposited crystals act as a hindrance to the application of important characterization techniques (e.g., mass loss measurement for the calculation of corrosion rate and examining the adhesion of the coating to metallic stents after fluid dynamic exposure). Therefore, to better characterize the coatings, the removal of these depositions is significant. In this work, we investigate the influence of in vitro test conditions in fluid dynamic biostability tests on the biostability of titanium oxynitride (TiO_X_N_Y_) coated stainless steel stents by adapting various fluid dynamic experimental parameters. The experimental conditions are based on modification in the components of fluid dynamic setup (e.g., tubings), simulated body fluid (SBF), with and without Ca^++^ and Mg^++^ ions, and the cleaning procedure (use of water, acetone, and isopropanol). Four different experiments were conducted under various experimental parameter sets. SEM and EDX measurements were used for the identification of degradation products after each experiment. This study highlights the importance of optimized experimental conditions showing negligible depositions when utilizing Puriflex tubing or a comparable artificial vessel, SBF devoid of Ca^++^ and Mg^++^ ions, and performing sample cleaning with distilled water in an ultrasonic bath. The presented conditions were optimized for titanium oxynitride coated samples. A similar approach could be applied to other samples with or without some small variation.

## 1. Introduction

Stents are small, tubular implants that are commonly used to treat atherosclerosis, e.g., coronary artery disease. After being implanted, the stent needs to provide the required mechanical strength, sufficient resistance to corrosion, and adequate biocompatibility. Generally, stents can be permanent, temporary, and drug eluting. Permanent stents are metallic stents that are also known as bare metal stents (BMS). So far, Tantalum (Ta), alloys of platinum-iridium (Pt-Ir), cobalt-chromium (Co-Cr), stainless steel (316L), and nitinol (Ni–Ti) are the materials utilized to make metallic stents [1]. The risk of in-stent restenosis, tissue hyperplasia, and explantation (in some cases) in BMS leads to the development of coated metallic stents [2,3]. Due to the complex geometrical shape of stents, the uniform coating coverage on stents is, however, still a challenging task, and so is the evaluation of coating delamination and degradation after implantation [4]. Therefore, strategies to perform a careful evaluation of coating during biostability and degradation tests are of equal importance.

Assessing the in vitro biostability of coated stents relies heavily on mass loss and corrosion measurements [5,6,7]. During degradation tests, in addition to the deterioration of the coating or substrate material, degradation products also form on the sample surface. A deposition-free degradation process is essential to closely examine the coating itself and to facilitate precise measurements of mass loss and corrosion.

Both in vitro and in vivo studies on stents have shown the formation of salt deposits or crystallization on their surfaces [8,9]. The nature and chemical composition of these deposits largely depends on the stent material and the composition of the surrounding fluid [10].

In fluid dynamic experiments, degradation is controlled by varying factors such as pressure, flow rate, flow velocity, shear stress, viscosity, and temperature. Additionally, the degradation products on the implant surface are influenced by the experimental setup and the type of fluid used [11,12]. Moreover, cleaning procedures [13] to remove the degradation products are also significant in order to prevent any damage (e.g., crack formation, chemical breakdown, delamination, etc.) to the implant surface.

Augthun et al., 1998 [13] conducted in vitro studies to examine the impact of cleaning methods on various implant surfaces. They found that cleaning procedures could potentially damage the implant surface and that if cleaning is incomplete, it becomes difficult to assess the extent of damage caused by the method.

For cleaning the substrate materials, the use of acetone, ethanol, isopropanol, and water in an ultrasonic bath has been reported in many studies [14,15,16,17]. However, for cleaning coated samples, mostly only water is used to avoid any potential damage to the coated surface due to cleaning.

To date, no studies have been reported that optimize the experimental setup and conditions for coated stents to assess their impact on stent degradation. This work aims to develop a strategy for optimizing conditions to obtain less deposition and degradation in coated stents.

In our previous work [8], we presented the fluid dynamic biostability test on titanium oxynitride (TiO_X_N_Y_) coated stainless stents. After 1 week of dynamic exposure to simulated body fluid, the surface of the stent was covered with heavy and low salt depositions, and therefore, no quantitative degradation analysis was possible. The present study was conducted as a continuation of our previous research with different testing strategies to evaluate the coating degradation behavior of TiO_X_N_Y_ coated stents during in vitro biostability tests.

In this work, TiO_X_N_Y_ coated stents and planar surfaces were used as test samples. Different strategies were adopted to optimize the experimental procedure. Our results show the significance of experimental conditions and cleaning procedures. The experimental conditions lead to unnecessary depositions on the implant surface during the degradation experiment, and cleaning procedures may lead to damage to the implant surface.

Therefore, to achieve deposition-free degradation on the stent surface, four experimental parameter sets were employed by varying the duration, tubing materials, and fluids. Additionally, two cleaning procedures involving distilled water, acetone, and isopropanol were applied to the coated stents.

The current work discusses the influence of applied experimental conditions and cleaning procedures on the formation of degradation products. The results are supported by the microscopy and SEM-EDX measurements.

## 2. Materials and Methods

### 2.1. Fluids

The chemical composition of the simulated body fluids used in this study is summarized in Table 1. For ease of reference, SBF containing Ca^++^ and Mg^++^ ions is referred to as SBF^++^, SBF without Ca^++^ and Mg^++^ ions as SBF^−−^, and Hanks Balanced Salt Solution (HBSS) without Ca^++^ and Mg^++^ ions as HBSS^−−^.

### 2.2. Samples and Coating

Medical grade 316L stainless steel stents and flat samples of 10 mm in diameter and 2 mm in height were used. The composition of the used stainless steel is presented in Table 2. All the samples were coated via pulsed reactive magnetron sputtering in two different methodologies, i.e., A and B. Table 3 summarizes the samples used in the experiments.

#### 2.2.1. Coating Methodology A

The deposition method used reactive magnetron sputtering through a UVN-200MI medium-frequency setup, which included a 200 mm diameter disk magnetron built up as a monoblock that contained the cathode and box-anode assemblies. In order to carry out this procedure, a reactive gas mixture consisting of argon, nitrogen, and oxygen was introduced into the vacuum chamber under ion bombardment conditions. The substrates were subjected to a negative bias voltage of −200 V in order to facilitate the deposition of coating ingredients from the gas plasma. Using titanium as the target material, a discharge power of 1 kW, a current of 3 A, a total gas pressure of 1.9 × 10^−1^ Pa, and an argon flow rate of 1.4 mL/min as the plasma-forming gas were among the particular parameters for deposition. With duty cycles ranging from 50% to 90% and pulsating frequencies ranging from 10 kHz to 60 kHz, the frequency was set at 20 kHz. A layer thickness between 150 and 170 nm was achieved by supplying the gas at a rate of 5 mL/min.

#### 2.2.2. Coating Methodology B

The process of pulsed reactive magnetron sputtering is a proven method for thin oxide layer deposition. We developed nitrogen-containing titanium oxide (N-TiO_2_) thin films in this study using the UVN-200MI vacuum pulsed magnetron sputtering machine (VIP Technologies LTD, Nizhny Novgorod, Russia). A 200 mm flat cathode is included in a single block with a body-anode and cathode assembly in a disk magnetron sputtering system. The deposition was done in a mixed environment of nitrogen, oxygen, and argon while samples were positioned on a rotating table 100 mm away from a titanium VT1-0 target.

The samples were first cleaned in an ultrasonic bath using an alcohol solution of toluene for 10 min as part of a predetermined protocol for the deposition process. Following their transfer into the vacuum chamber at a pressure of 10^−3^ Pa, they were treated with Ar plasma for 10 min at a pressure of 0.5 Pa using a discharge current of 3.0 A and a negative bias of −400 V.

The following phase involved using a discharge power of 0.5 kW and a negative bias of −200 V at a frequency of 20 kHz to deposit a titanium sublayer for 5 min in an argon environment at a pressure of 0.2 Pa. With a negative bias of −150 V and a frequency of 20 kHz, the process then switched to oxide mode by raising the power to 1 kW and adding oxygen and nitrogen, enabling the deposition of TiO_X_N_Y_ over a period between 3 and 5 min.

In the last stage, the TiO_X_N_Y_ layer was deposited a total gas pressure of 1.9 × 10^−1^ Pa (0.2 Pa) and an argon pressure of 1.25 × 10^−1^ Pa; the discharge power was kept constant at 1 kW. After 45 min of deposition, the samples’ negative bias stayed at −150 V and a frequency of 20 kHz, producing a coating thickness between 150 and 170 nm.

### 2.3. Fluid-Dynamic Experiments

The experiments were conducted at a temperature of 37 °C, with a flow velocity of 180 cm/s and a regulated pH of 7.4. The utilized artificial blood plasma was the simulated body fluid described in Beshchasna et al. [8] and Hanks Balanced Salt Solution (Lonza, Walkersville, MD, USA, without Ca^++^ and Mg^++^ ions). Silicon and Puriflex tubes were utilized to mimic artificial blood vessels. Fluid dynamic tests were performed for durations of 7 and 30 days to evaluate the degradation behavior of TiO_X_N_Y_ coated stents.

**Figure 1:**
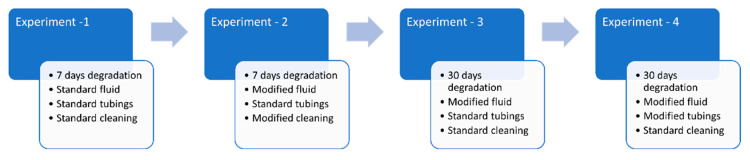
Schematic diagram of the experimental plan.

Figure 1 shows the general experimental plan for the present study; the standard fluid is SBF^++^, modified fluids are SBF^−−^ and HBSS^−−^, standard tubings mean regular silicon tubes, modified tubings are Puriflex tubes, standard cleaning means cleaning with only distilled water in the ultrasonic cleaning bath, the modified cleaning means cleaning with water, isopropanol, acetone in an ultrasonic bath. The following experiments were carried out to optimize the conditions for achieving deposition-free degradation:

Experiment no. 1: a 1 week fluid-dynamic experiment using a silicon tube and SBF^++^.
Experiment no. 2: a 1 week fluid-dynamic experiment using a silicon tube and SBF^−−^.
  1. Cleaning with water.
  2. Cleaning with water, isopropanol, and acetone.
Experiment no. 3: 30 days fluid-dynamic experiment using a silicon tube and HBSS^−−^.
Experiment no. 4: 30 days fluid-dynamic experiment using a Puriflex tube and HBSS^−−^.

### 2.4. Fluid-Dynamic Testing Setup

**Figure 2:**
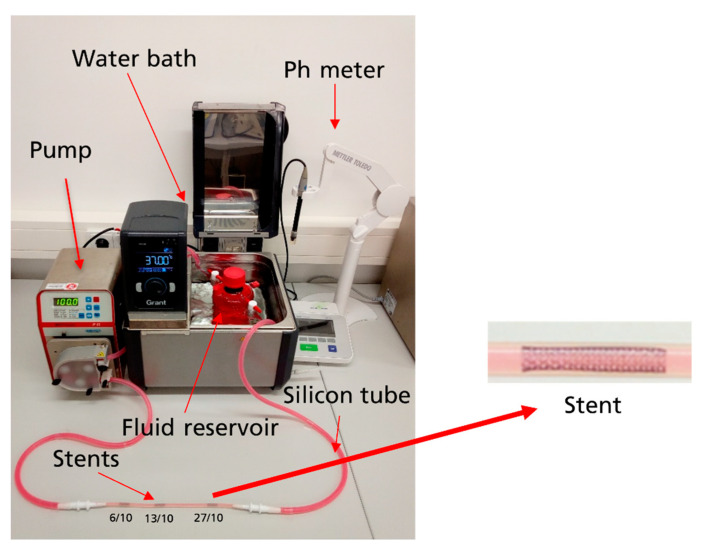
Fluid dynamic experimental setup for experiment 3. The setup contains a pump (to replicate fluid flow inside the arteries); a water bath (heat circulating water bath to regulate the temperature); a silicon tube (artificial vessel with an 8 mm inner diameter for fluid flow and a 3 mm inner diameter for stents); stents (TiO_X_N_Y_ (O_2_/N_2_ = 6/10, 13/10, 27/10); fluid reservoir (glass container with HBSS^−−^); and Ph meter (to monitor the pH levels during the experiment). The red color indicates the presence of phenol red in HBSS^−−^. It was used as a pH indicator.

The in vitro biostability of samples was evaluated using the fluid-dynamic experimental setup presented in Saqib et al. (2021 and 2024) and Beshchasna et al. [6,8,18]. The setup contains the peristaltic pump of type MCP Process IP65 (ISMATEC GmbH, Grevenbroich, Germany), heat circulating water bath, artificial vessels, and sensors for controlling temperature, flow velocity, and pH values. This system was employed with different types of simulated body fluid, artificial vessels, experiment durations, and cleaning methods. Figure 2 shows the fluid dynamic experimental setup for experiment no. 3, where silicon tubes and HBSS^−−^ (Lonza, Walkersville, MD, USA) with phenol red were used.

### 2.5. Surface Morphology

The surface morphology was evaluated using SEM-EDX and optical microscopy. The XL30 ESEM FEG Environmental Scanning Electron Microscope (Philips, Amsterdam, Netherlands) was used to take all of the SEM images and EDX analysis of the flat and stent samples. For SEM, a spot size of 3 nm and an accelerating voltage of 3.0 kV were set up. For EDX, the spot size was set to 5 nm, and the accelerating voltage was set to 10.0 kV.

A digital microscope VHX-X1 (Keyence Corporation, Osaka, Japan) was used for optical microscopic images of samples.

## 3. Results and Discussion

### 3.1. Experiment No. 1: 1 Week Fluid-Dynamic Experiment Using a Silicon Tube and SBF^++^

Firstly, TiO_X_N_Y_ coated stainless steel planar samples and stents with three different O_2_/N_2_ ratios (1:2, 1:5, 1:10) were subjected to 7 days of degradation. In this series of samples, the adhesion of the coating on the stents was found to be insufficient, as reported by Beshchasna et al. [8], while the planar samples exhibited a uniform coating across their surfaces.

**Figure 3:**
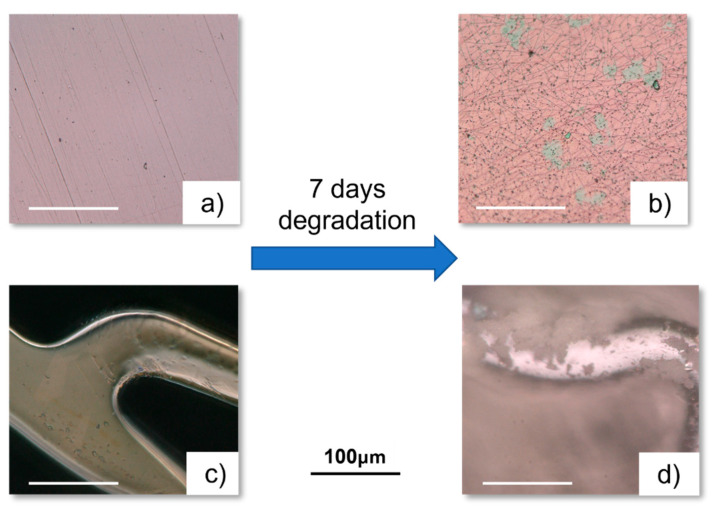
Microscopic images of TiO_X_N_Y_ planar (top) and stent (bottom) samples. (**a**,**c**) Samples before degradation, and (**b**,**d**) samples after 7 days of degradation under fluid dynamic loading.

Even though the degradation products were similar across all samples, the deposition form differed between the planar samples and the stents. In the planar samples, the deposition was in the form of cracked sheets, while in the stents, it appeared as crystallization (Figure 3).

**Figure 4:**
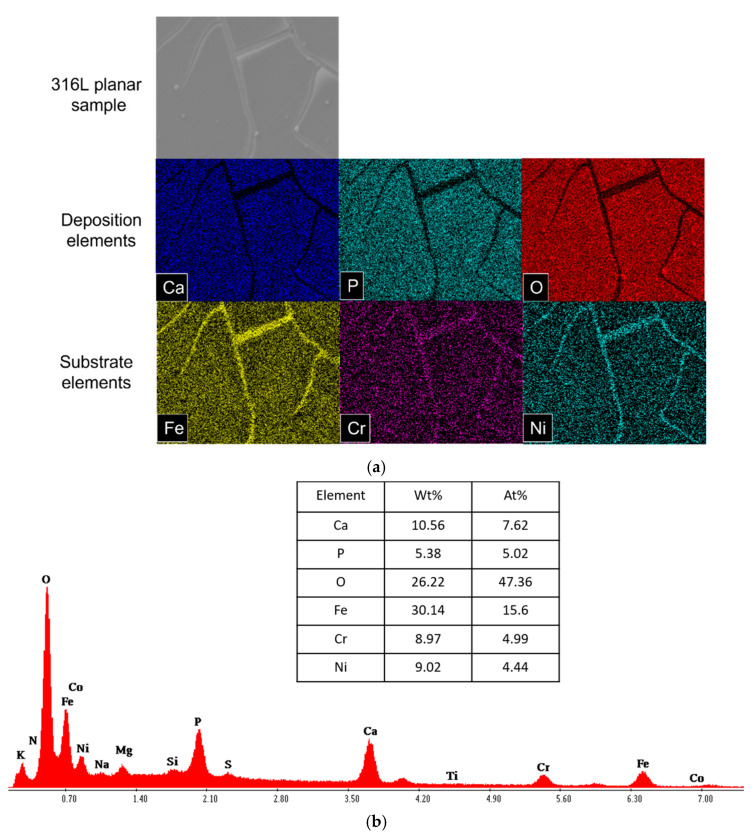
(**a**) Elemental mapping of the degradation products in the 316L planar sample through EDX analysis. (**b**) EDX analysis of planar 316L sample. EDX spectrum of the surface (shown in (**a**)) with elemental quantification.

**Figure 5:**
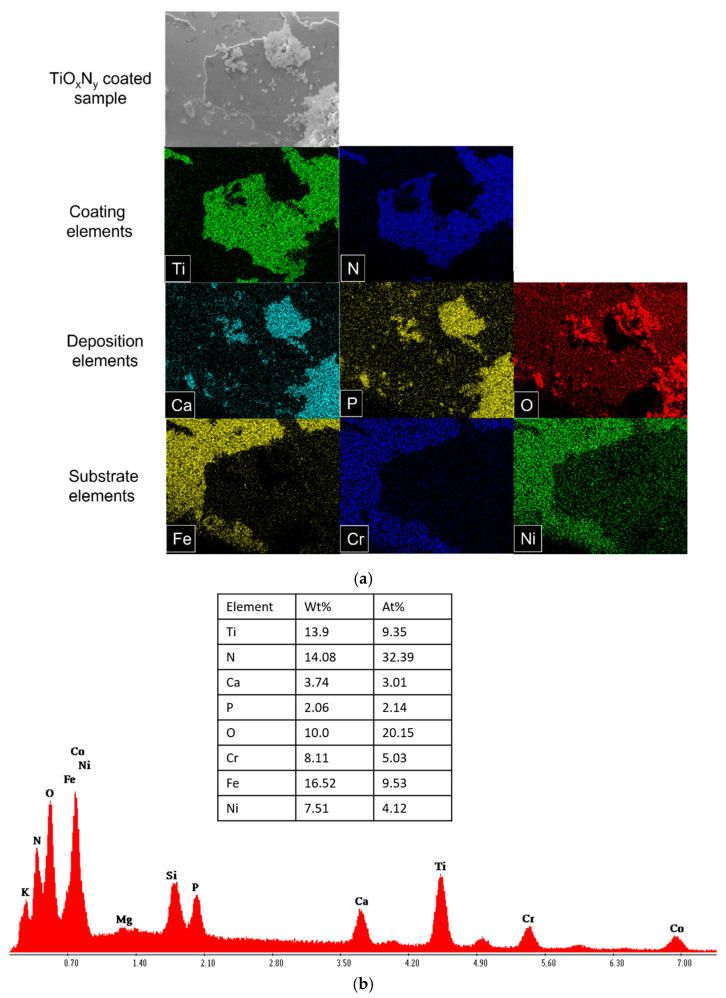
(**a**) EDX analysis of TiO_X_N_Y_ (O_2_/N_2_ = 1/10) coated stainless steel stent. Elemental mapping of degradation products. (**b**) EDX analysis of TiO_X_N_Y_ (O_2_/N_2_ = 1/10) coated stainless steel stent. EDX spectrum of the surface (shown in (**a**)) with elemental quantification.

Although the coating adhesion was inadequate, it can be inferred that the TiO_X_N_Y_ coating does not significantly affect the nature of the depositions. This is supported by the observation that 316L samples displayed similar degradation products to the TiO_X_N_Y_ coated samples (Figure 4 and Figure 5). After 7 days of degradation, all planar samples exhibited cracked surfaces. Research groups investigating similar surfaces for biomimetic coatings [19,20] have also reported the occurrence of these cracked deposition layers on TiO_2_ surfaces.

Figure 4a illustrates the formation of hydroxyapatite after the degradation period. Elemental mapping of the degradation products revealed the presence of Ca/P. Similar degradation products were observed in all other planar samples. Additionally, the prominent peaks of Ca, P, and O in Figure 4b confirm the formation of Ca/P hydroxyapatite crystals on the sample surface. Moreover, elemental quantification further highlights their significant presence.

The upper surface of the stents, which was in contact with the silicon tube, did not directly interact with the simulated body fluid. Consequently, degradation products were found on the remaining three sides of the stent strut, especially on the inner side, which was exposed to the flowing SBF.

Figure 5a presents the elemental mapping of the upper surface of the TiO_X_N_Y_ coated stent after 7 days of degradation. Similar to the planar samples, the stent samples also showed the presence of Ca, P, and O in the degradation products. Clear peaks of hydroxyapatite elements were visible in the stent samples (Figure 5b), with a notable presence in both wt% and at%.

The hydroxyapatite crystallization is likely due to the hydrophilic nature of TiO_2_, which tends to form titanium hydroxide groups [20], when exposed to SBF [21]. These Ti-OH groups promote the nucleation and crystallization of apatite, as noted by Toshihiro et al. [20].

### 3.2. Experiment No. 2: 1 Week Fluid-Dynamic Experiment Using a Silicon Tube and SBF^−−^

#### 3.2.1. Cleaning with Water

**Figure 6:**
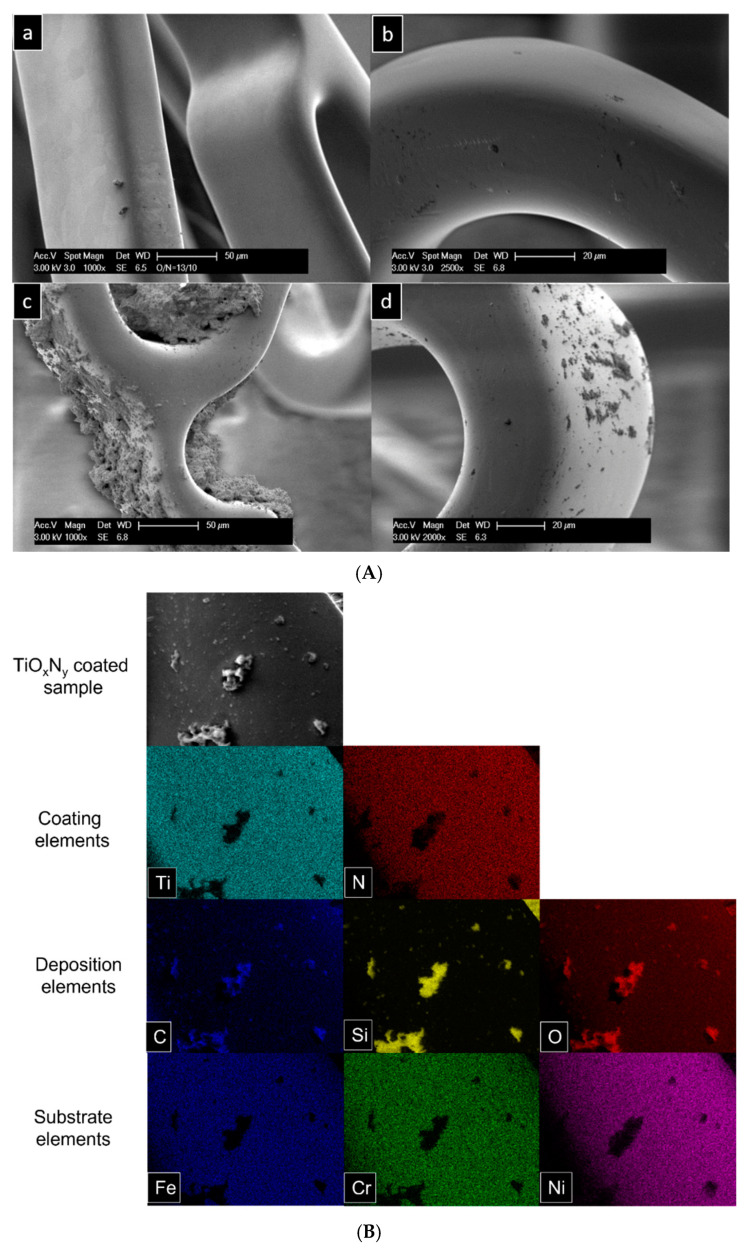

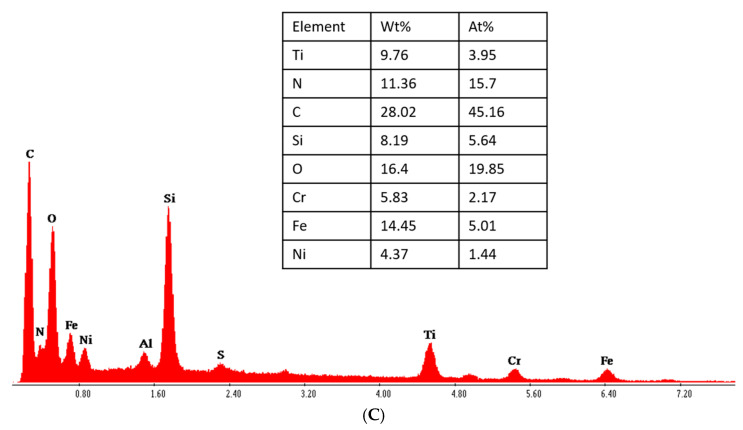
(**A**) Stent surface before and after degradation test. (**a**,**b**) Undegraded stent surface, and (**c**,**d**) 7 days degraded stent surface. (**B**) EDX analysis of TiO_X_N_Y_ (O_2_/N_2_ = 27/10) coated stainless steel stent. Elemental mapping of degradation products. (**C**) EDX analysis of TiO_X_N_Y_ (O_2_/N_2_ = 27/10) coated stainless steel stent. EDX spectrum of the surface (shown in (**B**)) with elemental quantification.

All stent samples were characterized via SEM-EDX measurements before being exposed to simulated blood plasma under physiological conditions. The surface morphology of the stents, coated with TiO_X_N_Y_ at O_2_/N_2_ ratios of 3:5, 13:10, and 27:10, showed strong coating adhesion to the substrate with little to no visible pits. Figure 6 demonstrates that the coating quality was noticeably better compared to the samples from earlier experiments. No delamination was observed, although some pits were occasionally present on the stent surfaces. In previous experiments, significant calcium phosphate deposition was observed on the stents after exposure to simulated blood plasma. To address this issue, other research groups have used simulated blood plasma without Ca and Mg ions to avoid such deposits and improve coating characterization [10,22,23,24]. Similarly, in this study, simulated blood plasma without Ca and Mg ions was employed to prevent unnecessary deposit formation.

The improved coating quality of the stents was expected to provide greater resistance to delamination and degradation in aggressive biological conditions compared to the previous stents. Additionally, the exclusion of Ca and Mg ions from the SBP was intended to prevent deposit formation. After 7 days of exposure to SBP, the samples underwent cleaning in distilled water for 5 min in an ultrasonic bath. However, SEM analysis indicated that despite the coatings enduring the harsh biological medium, depositions were still present on all three stents.

Notably, the deposition levels were found to differ across various sides of the stent, showing a nonuniform pattern (Figure 6A). Elemental mapping of the deposited regions identified C, Si, and O as the primary components. As anticipated, due to the modified simulated blood plasma, Ca and P were absent in the deposition crystals. Surprisingly, however, C, Si, and O were consistently detected across all three samples (Figure 6B). Additionally, the EDX spectra from the same area revealed pronounced peaks for C, Si, and O, with no detectable peaks for Ca, P, or Mg (Figure 6C).

The same stents were examined with SEM before the degradation test. As a result, the Si and Al peaks are attributed to remnants of the carbon adhesive tabs, which contain Si, Al, O, and C elements [4]. These elements are part of the carbon tabs used to attach the stents to the sample holder for the SEM-EDX analysis. Removing Ca and Mg ions did not resolve the deposition problem. Therefore, the samples were cleaned with acetone, isopropanol, and distilled water to remove the deposits.

#### 3.2.2. Cleaning with Water, Isopropanol, and Acetone

**Figure 7:**
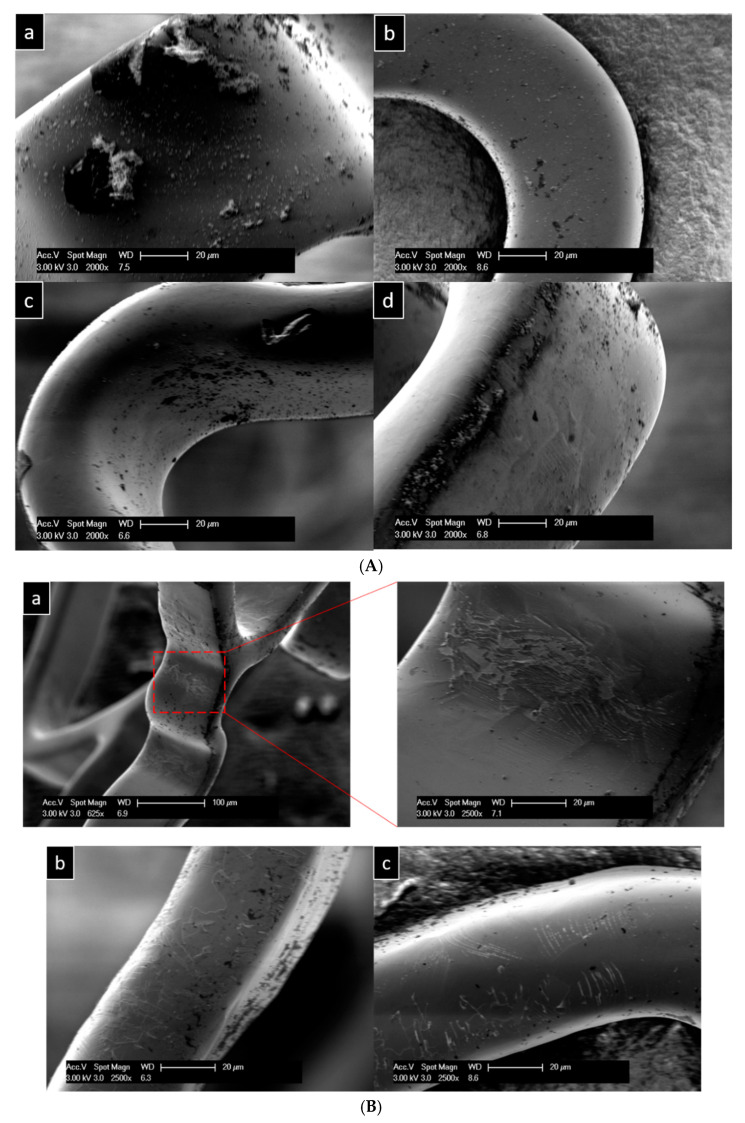

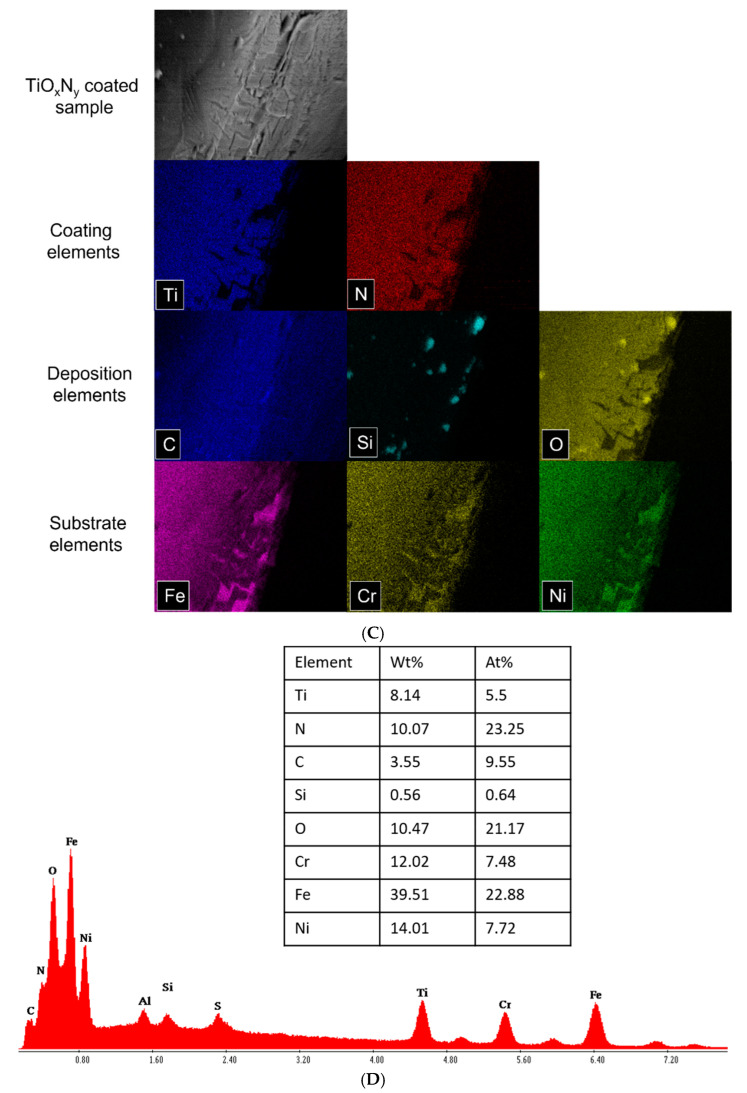
(**A**) Stent surface after cleaning with water, isopropanol, and acetone in ultrasonication. (**a**) Upper surface, (**b**) inner surface, (**c**) right surface, and (**d**) left surface of the stent wire. (**B**) Damaged stent surfaces. (**a**) Curved surfaces, (**b**,**c**) flat surfaces. (**C**) Elemental mapping of the damaged stent surface. (**D**) EDX spectrum of the damaged stent surface with the quantification of the elements.

Figure 7A illustrates the presence of depositions on all sides of the stent strut, indicating that cleaning with isopropanol and acetone had no effect. This cleaning method led to two issues: (i) it failed to remove the depositions, and (ii) it caused damage to the coating (Figure 7B). As seen in Figure 7B, the coating was compromised in all areas of the stent wire. Additionally, elemental mapping of one of the damaged regions revealed the absence of coating elements (Figure 7C). The degradation products observed after this cleaning protocol were similar to those found when using water. The EDX spectrum from the surface, shown in Figure 7C, along with the element quantification, further confirms the presence of degradation products such as C, Si, and O (Figure 7D).

The cleaning method involving isopropanol, acetone, and water failed to remove the depositions and resulted in coating damage. In contrast, the studies referenced earlier, which used similar cleaning protocols [14,15,16,17], did not apply the same procedures to their coated samples, instead opting to clean with only water. However, Orinakova et al. [16] did employ a comparable cleaning procedure for HAp and MnHAp coated Fe samples. These surfaces showed uneven coating distribution and cracks. It is possible that the aggressive cleaning method did not cause further damage to their coated samples, or the damage may have been difficult to observe. The effectiveness of a cleaning procedure is highly influenced by the surface chemistry of the coated material, making water-based cleaning the safest option for preserving the integrity of coated surfaces and preventing damage from harsh chemicals.

### 3.3. Experiment No. 3: 30 Days Fluid-Dynamic Experiment Using a Silicon Tube and HBSS^−−^

After observing unwanted depositions (C, Si, and O) in the second experiment, likely caused by the carbon adhesive tabs, the experiment was repeated with new stents for 30 days. This was done for two main reasons: (i) to assess the durability of the coatings over a longer degradation period and (ii) to eliminate the depositions from the carbon adhesive tabs, thereby allowing the stents to be suitable for mass loss measurements.

**Figure 8:**
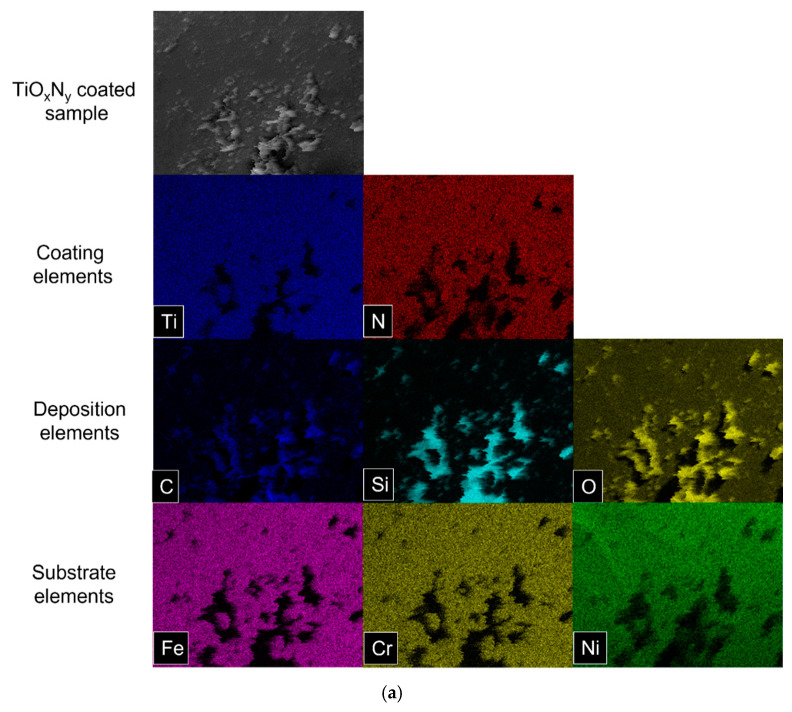

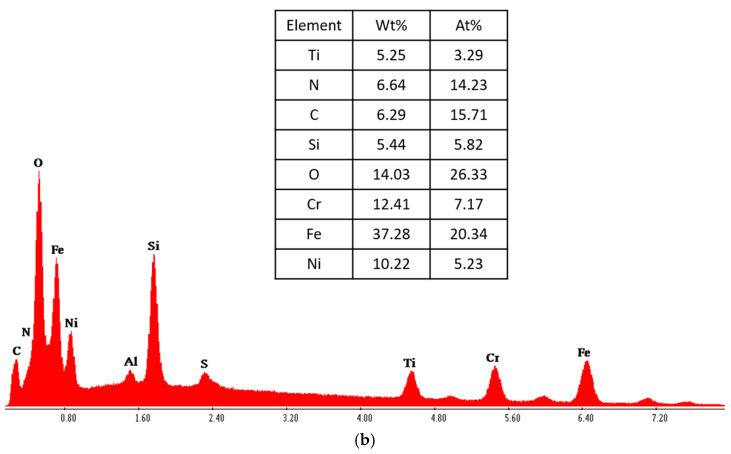
(**a**) EDX analysis of TiO_X_N_Y_ (O_2_/N_2_ = 6/10) coated stainless steel stent. Elemental mapping of degradation products. (**b**) EDX analysis of TiO_X_N_Y_ (O_2_/N_2_ = 6/10) coated stainless steel stent. Elemental mapping of degradation products.

Interestingly, results similar to those observed in the previous experiment were obtained. Figure 8a displays the elemental mapping of the degradation products on the stent surface. The degradation products showed the same elements—C, Si, and O. However, the carbon peak is noticeably lower compared to the second experiment (Figure 8b), likely due to the absence of contact with the carbon adhesive tabs. The peaks remain similar to those observed in the second experiment. These findings suggest that there is another factor contributing to these depositions beyond the carbon adhesive tabs. Based on these observations, Puriflex tubes were used in the next experiment to replace the silicon tubes.

### 3.4. Experiment No. 4: 30 Days Fluid-Dynamic Experiment Using a Puriflex Tube and HBSS^−−^

**Figure 9:**
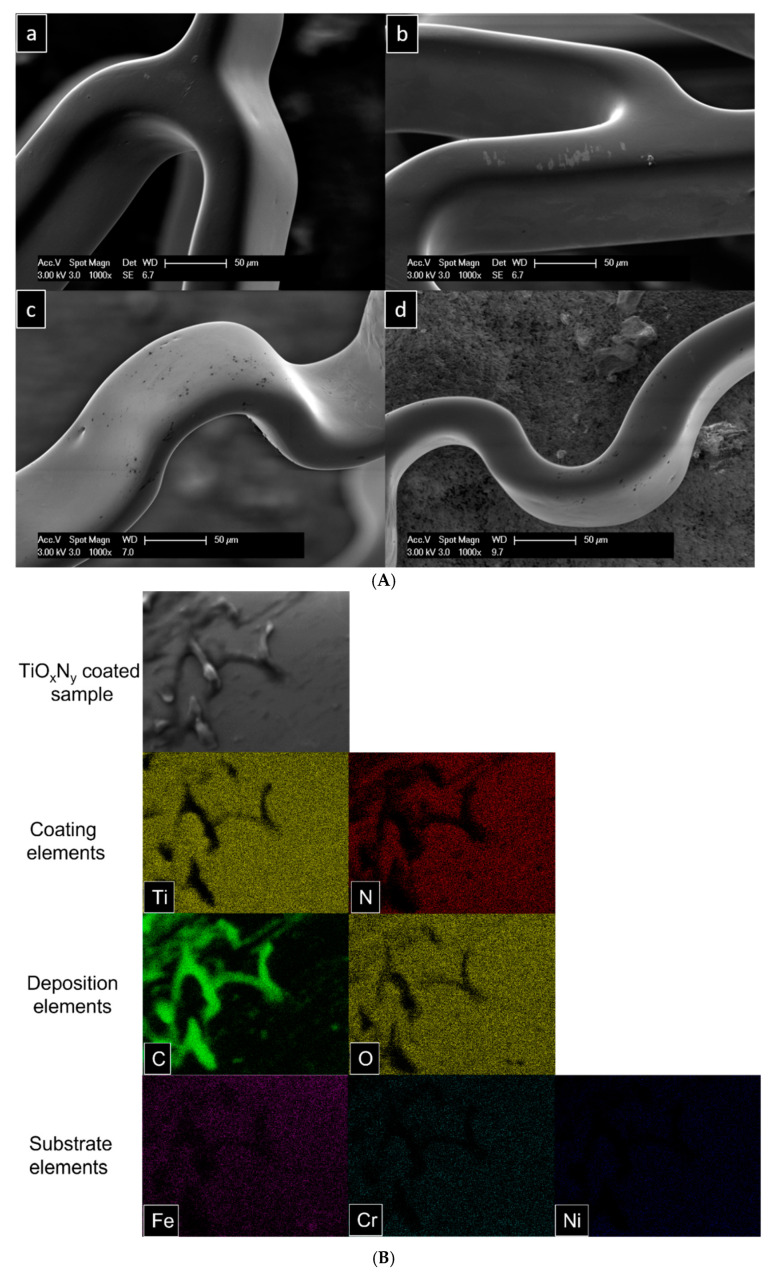

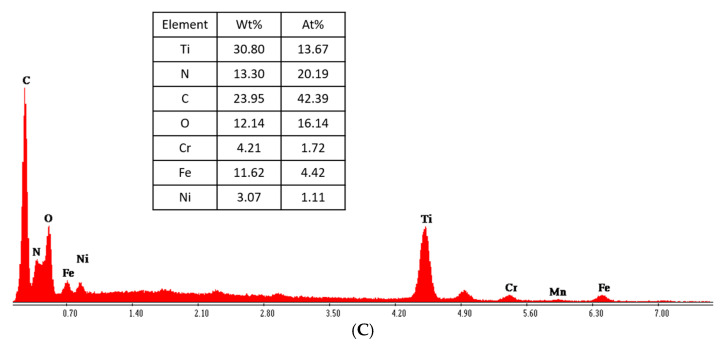
(**A**) Stent surface after cleaning with water in ultrasonication. (**a**,**b**) Stent surface from the branched geometrical part, and (**c**,**d**) stents surface from the curled geometrical part. (**B**) EDX analysis of TiO_X_N_Y_ (O_2_/N_2_ = 1/1) coated stainless steel stent. Elemental mapping of degradation products. (**C**) EDX analysis of TiO_X_N_Y_ (O_2_/N_2_ = 1/1) coated stainless steel stent. Spectrum of surface (**B**) with elemental quantification.

In this experiment, different O_2_/N_2_ ratios (1:1, 2:1, 2:3) were tested. The coatings used displayed a biostable response after 30 days of degradation. Figure 9A illustrates the stent surface before and after the degradation process. With the use of Puriflex tubes in this experiment, negligible depositions were observed on the stent surface.

The deposition, in this case, only contained carbon, as shown in the elemental mapping of the EDX analysis (Figure 9B). The EDX spectra also confirm the presence of only a carbon peak aside from the coating and substrate materials (Figure 9C). The presence of carbon alone on the stent surface suggests that no chemical reaction occurred between the coating material and the fluid. The minimal depositions could likely be attributed to dust in the environment, as the experiments were carried out in a standard laboratory environment.

**Figure 10:**
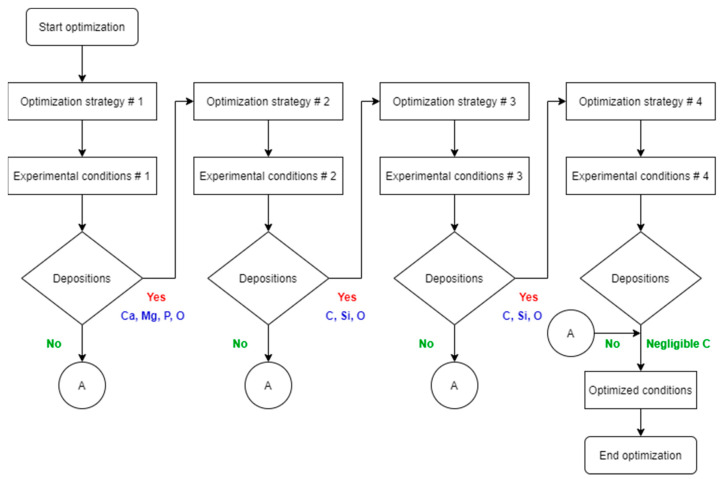
Schematic overview of the optimization process.

Figure 10 summarizes the optimization process to get the deposition-free fluid dynamic biostability tests to evaluate the coating degradation throughout the sample surface. It summarizes the presence or absence of degradation products and the elements present in the degradation depositions.

In the presented experiments, the physiological temperature of 37 °C was used, while a high flow velocity of 180 cm/s (five times higher than the flow velocity of arteries) was also used to accelerate the degradation process. It is important to consider that changes in flow rates can have a significant effect on ion transport, the formation of degradation products, and the stability of the coating.

Despite the fact that the authors have addressed the issue of depositions, negligible depositions were observed after the degradation tests with the fourth optimization strategy. However, there are various limitations of this study that need to be considered when optimizing it further or for other samples.

The contamination was observed on the sample surface, which could have occurred due to the experiments and sample handling in a standard lab environment. This can be avoided in a clean room environment from the sample preparation until the last characterization.

In this study, only TiO_X_N_Y_ coated 316L samples were used. It is difficult to predict the response of the other coating or substrate to a similar kind of setting.

Another limitation of this study is that our results are based on single measurements, as repeated experiments were not feasible due to the high experimental costs (e.g., stents). While this approach limits the ability to evaluate the repeatability of the findings, the primary goal of the research was to qualitatively assess the influence of experimental conditions and various parameters during fluid dynamic degradation tests. To ensure the reliability of the data, we carefully controlled the experimental conditions by calibrating the equipment, following standardized testing protocols, and consistently monitoring all test parameters.

The authors acknowledge that the absence of repeated measurements may restrict the broader applicability of the findings. Future studies should aim to include repeated experiments where possible to strengthen the validation of observed facts and further confirm the conclusions. Despite this limitation, the outcomes of this study provide meaningful insights into the degradation mechanisms of coated metallic stents in various experimental conditions and offer valuable guidance for designing future experiments.

Despite using different cleaning strategies, the authors could not safely remove the degradation products from the sample surface. Therefore, it is still an open question for future research works.

In general, the authors provide the following suggestions based on their experience:

1. The experimental environment should be controlled and cleaned so that no contamination should occur.
2. The tubes should be resistant to the used fluids and fluid pressure.
3. For every experiment, sterilized new tubes (and connectors) should be used to avoid any contamination from previous experiments.
4. Cleaning the sensors in an effective way to make sure that there are no traces of degradation products from previous experiments.
5. In a real physiological environment, the samples are exposed to Ca and Mg ions, but to avoid degradation products and to see the whole coated area, fluid without these can be used. However, the fluids with ions should be used separately to evaluate the degradation of the coated sample in the presence of all possible ions.

## 4. Conclusions

TiO_X_N_Y_ coatings demonstrate resistance to aggressive biological media, resulting in a notably biostable response. The O_2_/N_2_ ratio affects the extent of depositions and degradation products but does not alter their chemical composition. Both 316L stainless steel and TiO_X_N_Y_ samples exhibit similar degradation products during in vitro fluid dynamic degradation tests. Various experimental and cleaning methods were employed to remove these depositions. Our findings reveal that cleaning TiO_X_N_Y_ coated samples with distilled water in an ultrasonic environment does not damage the coatings, although it fails to eliminate degradation products. Additionally, cleaning with acetone, isopropanol, and distilled water in an ultrasonic bath does not remove the degradation products and results in coating damage. Simulated blood fluids containing Ca^++^ and Mg^++^ ions promote the deposition of Ca^++^ and Mg^++^ phosphates on the stent surface, while silicon tubes contribute to Si deposition. The optimal approach for conducting fluid dynamic experiments on TiO_X_N_Y_ stents to achieve deposition-free degradation involves using Puriflex tubing or a similar artificial vessel, SBF without Ca^++^ and Mg^++^ ions, and cleaning the samples with distilled water in an ultrasonic bath.

## Figures and Tables

**Table 1:** Fluid composition.

Composition	Concentration (g/L)		
	SBF^++^	SBF^−−^	HBSS^−−^
NaCl	6.800	6.800	8.000
KCl	0.400	0.400	0.400
MgSO_4_	0.100	-	-
CaCl_2_	0.200	-	-
NaHCO_3_	2.200	2.200	0.350
Na_2_HPO_4_	0.130	0.130	0.009
NaH_2_PO_4_	0.030	0.030	-
KH_2_PO_4_	-	-	0.006
C_6_H_12_O_6_ (Dextrose)	-	-	1.000
C_19_H_14_O_5_S (Phenol red)	-	-	0.002

**Table 2:** Composition of the 316L stainless steel.

Element	Fe	Cr	Ni	Mo	Mn	Si	N	P	C	Si
%	balance	16–18	10–14	2–3	2	0.75	0.1	0.45	0.03	0.03

**Table 3:** Sample summary.

Experiment No.	Coating Method	Feeding Ratio (O_2_/N_2_)	Samples
		½	TiO_X_N_Y_ stents and planar samples
1	A	1/5	
		1/10	
		6/10	TiO_X_N_Y_ stents
2 and 3	B	13/10	
		27/10	
4		1/1	TiO_X_N_Y_ stents
	B	2/1	
		2/3	

## Data Availability

The original contributions presented in this study are included in the article. Further inquiries can be directed to the corresponding authors.

## References

[1] Saqib M. (2022). Application of Experimental and Analytical Approaches in Characterizing Coronary Stents. Ph.D. Thesis.

[2] Babapulle M.N., Eisenberg M.J. (2002). Coated Stents for the Prevention of Restenosis: Part I. Circulation.

[3] Buccheri D., Piraino D., Andolina G., Cortese B. (2016). Understanding and Managing In-Stent Restenosis: A Review of Clinical Data, from Pathogenesis to Treatment. J. Thorac. Dis..

[4] Hopkins C., Sweeney C.A., O’Connor C., McHugh P.E., McGarry J.P. (2016). Webbing and Delamination of Drug Eluting Stent Coatings. Ann. Biomed. Eng..

[5] (2010). Standard Practice for Calculation of Corrosion Rates and Related Information from Electrochemical Measurements.

[6] Saqib M., Kuzmin O., Kraskiewicz H., Wasyluk L., Cuniberti G., Ficai A., Pichugin V., Opitz J., Beshchasna N. (2021). Evaluation of in Vitro Corrosion Behavior of Titanium Oxynitride Coated Stainless Steel Stents. IEEE Access.

[7] (2012). Standard Practice for Laboratory Immersion Corrosion Testing of Metals.

[8] Beshchasna N., Ho A.Y.K., Saqib M., Kraśkiewicz H., Wasyluk Ł., Kuzmin O., Duta O.C., Ficai D., Trusca R.D., Ficai A. (2019). Surface Evaluation of Titanium Oxynitride Coatings Used for Developing Layered Cardiovascular Stents. Mater. Sci. Eng. C.

[9] Wang Z., Sun Z., Han B., Zheng Q., Liu S., Zhang B., Duan T. (2020). Biological Behavior Exploration of a Paclitaxel-Eluting Poly-l-Lactide-Coated Mg-Zn-Y-Nd Alloy Intestinal Stent: In Vivo. RSC Adv..

[10] Barrere F., Van Blitterswijk C.A., De Groot K., Layrolle P. (2002). Nucleation of Biomimetic Ca-P Coatings on Ti6Al4V from a SBF Â 5 Solution: Influence of Magnesium. Biomaterials.

[11] Tkacz J., Slouková K., Minda J., Drábiková J., Fintová S., Doležal P., Wasserbauer J. (2017). Influence of the Composition of the Hank’s Balanced Salt Solution on the Corrosion Behavior of AZ31 and AZ61 Magnesium Alloys. Metals.

[12] Li L.Y., Liu B., Zeng R.C., Li S.Q., Zhang F., Zou Y.H., Jiang H.G., Chen X.B., Guan S.K., Liu Q.Y. (2018). In Vitro Corrosion of Magnesium Alloy AZ31—A Synergetic Influence of Glucose and Tris. Front. Mater. Sci..

[13] Augthun M., Tinschert J., Huber A. (1998). In Vitro Studies on the Effect of Cleaning Methods on Different Implant Surfaces. J. Periodontol..

[14] Mo X., Qian J., Chen Y., Zhang W., Xian P., Tang S., Zhou C., Huang N., Ji H., Luo E. (2021). Corrosion and Degradation Decelerating Alendronate Embedded Zinc Phosphate Hybrid Coating on Biodegradable Zn Biomaterials. Corros. Sci..

[15] Park J.H., Olivares-Navarrete R., Baier R.E., Meyer A.E., Tannenbaum R., Boyan B.D., Schwartz Z. (2012). Effect of Cleaning and Sterilization on Titanium Implant Surface Properties and Cellular Response. Acta Biomater..

[16] Oriňaková R., Oriňak A., Kupková M., Hrubovčáková M., Markušová-Bučková L., Giretová M., Medveckỳ L., Dobročka E., Petruš O., Kalavskỳ F. (2015). In Vitro Degradation and Cytotoxicity Evaluation of Iron Biomaterials with Hydroxyapatite Film. Int. J. Electrochem. Sci..

[17] Barrère F., Van Der Valk C.M., Dalmeijer R.A.J., Van Blitterswijk C.A., De Groot K., Layrolle P. (2003). In Vitro and in Vivo Degradation of Biomimetic Octacalcium Phosphate and Carbonate Apatite Coatings on Titanium Implants. J. Biomed. Mater. Res. Part A.

[18] Saqib M., Kremmer K., Opitz J., Schneider M., Beshchasna N. (2024). Evaluation of the Degradation Properties of Plasma Electrolytically Oxidized Mg Alloy AZ31 Using Fluid Dynamic Accelerated Tests for Biodegradable Implants. J. Funct. Biomater..

[19] Jalota S., Bhaduri S.B., Tas A.C. (2006). Effect of Carbonate Content and Buffer Type on Calcium Phosphate Formation in SBF Solutions. J. Mater. Sci. Mater. Med..

[20] Kasuga T., Kondo H., Nogami M. (2002). Apatite Formation on TiO_2_ in Simulated Body Fluid. J. Cryst. Growth.

[21] Kokubo T. (1990). Surface Chemistry of Bioactive Glass-Ceramics. J. Non. Cryst. Solids.

[22] Hertel M., Laule M., Zinelis S., Imiolczyk S.M., Mueller W.-D. (2016). Electrochemical Characterization of Vascular Bare-Metal Stents. A Novel Approach Modifying the Mini-Cell System. SDRP J. Biomed. Eng..

[23] Lamaka S.V., Gonzalez J., Mei D., Feyerabend F., Willumeit-Römer R., Zheludkevich M.L. (2018). Local PH and Its Evolution Near Mg Alloy Surfaces Exposed to Simulated Body Fluids. Adv. Mater. Interfaces.

[24] Chen C., Tan J., Wu W., Petrini L., Zhang L., Shi Y., Cattarinuzzi E., Pei J., Huang H., Ding W.-J. (2018). Modeling and Experimental Studies of Coating Delamination of Biodegradable Magnesium Alloy Cardiovascular Stents. ACS Biomater. Sci. Eng..

